# Characteristics of intestinal microbiota in infants with late-onset breast milk jaundice

**DOI:** 10.3389/fnut.2023.1119768

**Published:** 2023-05-12

**Authors:** Qianying Guo, Xinran Liu, Mingxuan Cui, Xuening Li, Chen Yang, Shilong Zhao, Lina Pan, Xiaoyu Peng, Linlin Wang, Peng Liu

**Affiliations:** ^1^Department of Clinical Nutrition, Peking University People’s Hospital, Beijing, China; ^2^Department of Obstetrics and Gynecology, Peking University Third Hospital, Beijing, China; ^3^National Health Commission Key Laboratory of Reproductive Health, School of Public Health, Institute of Reproductive and Child Health, Peking University, Beijing, China; ^4^Ausnutria Dairy (China) Co., Ltd., Changsha, China

**Keywords:** late-onset breast milk jaundice, intestinal microbiota, 16S rRNA, breastfed infants, *Klebsiella*

## Abstract

**Introduction:**

In this paper, microbiota analysis was determined to analyze the structure and difference of intestinal microbiota between LBMJ (late-onset breast milk jaundice) infants and healthy individuals.

**Methods:**

We collected fresh fecal samples from 13 infants with LBMJ and 13 healthy individuals, then determined the intestinal microbiota by 16 s rRNA sequencing. The differences of microbiota structure, diversity and functional characteristics between the two groups were analyzed, and the correlation between dominant genus and TcB (transcutaneous bilirubin) value was calculated.

**Results:**

In this study, there were no significant differences in maternal demographic characteristics, neonatal status and macronutrients in breast milk between the two groups (*p* > 0.05). There are differences in the structure of intestinal microbiota between LBMJ and the control group. At the genus level, the relative abundance of *Klebsiella* in the case group is high (*p* < 0.05). At the same time, correlation analysis indicates that the abundance of *Klebsiella* is positively correlated with TcB value. The intestinal microbiota richness and diversity (Alpha diversity and Beta diversity) of the two groups were significantly different (*p* < 0.05). LEfSe analysis showed that 25 genera including *Klebsiella* was significantly enriched in the LBMJ infants, and the other 17 species are enriched in the control group. Functional prediction analysis indicated that 42 metabolic pathways may be related to the occurrence of LBMJ.

**Conclusion:**

In conclusion, characteristic changes are seen in intestinal microbiota compositions between LBMJ infants and the healthy controls. *Klebsiella* is closely associated with the severity of the disease, which may be due to enhanced β-glucuronidase activity.

## 1. Introduction

Hyperbilirubinemia is one of the most common conditions in newborns (1). Although it is generally considered to have a good prognosis, a large proportion of newborn patients maintain high bilirubin levels, which may lead to serious complications, such as growth retardation, encephalopathy, autism and hearing impairment.

LBMJ (late-onset breast milk jaundice) is a common type of hyperbilirubinemia, which occurs 1–2 weeks after the birth of breastfed infants, lasting 4–6 weeks or even 2–3 months. Breastfeeding is the biggest risk factor for LBMJ, but the etiology and mechanism are still unclear, and the diagnosis mainly depends on clinical exclusivity (2). Under these circumstances, interruption of breastfeeding to treat LBMJ is controversial and may increase the risk of early termination of breastfeeding which also contradicts the WHO (World Health Organization) recommendation of exclusive breastfeeding for 6 months (3).

Few studies have been reported on the effect of dietary factors of lactating mothers on LBMJ. A study on the effect of a Mediterranean diet on serum bilirubin levels showed that long-term adherence to this dietary pattern reduced serum bilirubin concentrations, but this study did not analyze the effect of specific food or nutrients in the diet on bilirubin metabolism (4). Loprinzi and Mahoney studied the effect of consumption of flavonoid-rich fruits and vegetables on serum bilirubin levels and found a positive correlation between them (5). In addition to the direct effect of diet on bilirubin metabolism, it is unclear whether dietary factors can contribute to the development of LBMJ by affecting the composition of breast milk. Gut microbiota is critical for bilirubin metabolism. Studies have shown that the composition of intestinal microbiota is closely related to the serum bilirubin level (6). Intestinal microbiota participates in bilirubin metabolism, while the disorder of intestinal microbiota interferes with the transformation of bilirubin, which may lead to the increase of bilirubin intestinal and liver circulation, leading to hyperbilirubinemia. Previous studies have found a relationship between *Clostridium*, *Bifidobacterium*, and bilirubin metabolism, but the role of microbiota induced neonatal bilirubin is not fully understood (7). Using germ-free multidrug resistance 2 knockout mice model, the researchers found that the absence of intestinal microbiota would aggravate the hepatobiliary disease of mouse models, proving the importance of symbiotic microbiota and its metabolites in preventing biliary injury (8). A randomized controlled trial has found that prebiotic oligosaccharides can reduce the level of bilirubin in premature infants and thus treat neonatal hyper-bilirubinemia (9). These results hint that infants with LBMJ may have abnormal intestinal microbiota, but its role in the development of LBMJ remains unclear.

We investigated the structure, diversity and difference of intestinal microbiota between LBMJ infants and controls on the 42nd day of birth to understand the possible mechanisms by which intestinal microbiota induces jaundice.

## 2. Materials and methods

### 2.1. Subjects

This study is based on a follow-up mother-infant cohort from the Peking University People’s Hospital.

Infants and their mother were screened and enrolled in the study based on the following inclusion criteria: Full-term infants born at Peking University People’s Hospital; The mother is 20–45 years old; Exclusive breastfeeding or mainly (breast feeding ac-counts for more than 80%); presenting with skin or sclera jaundice and do not subside for more than 3 weeks; Transdermal bilirubin value was greater than 7.87 mg/dL on the 42nd day of birth.

Infants were excluded for the following reasons: with risk factors including hemolytic disease, blood type incompatibilities, reticulocytosis, abnormal blood smear, polycythemia, Coombs’ test positive, glucose-6-phosphate dehydrogenase deficiency, skull hematoma, hypothermia, intracranial hemorrhage, cholestasis, neonatal hypothyroidism, phenylketonuria screening positive and used antibiotics and probiotics.

Finally, 13 infants with LBMJ were selected in case group and 13 healthy infants were included in control group. The study was approved by the Ethics Committee of the Peking University People’s Hospital (Approval Number: 2020PHB113-01), and all participating mothers signed an informed consent form.

### 2.2. Questionnaire survey

The questionnaire survey process by the training of qualified investigators to take a “face-to-face” questioning method to collect information, the collection of content includes mother’s maternal age, BMI, gestational week, delivery mode, parity, pregnancy complications, infant’s gender, birth length, birth weight, 1-min Apgar score and 5-min Apgar score.

The Apgar score is the standard method of assessing a child’s physical condition immediately after birth. It includes: muscle tone (Activity), pulse (Pulse), frowning movements response to stimuli (Grimace), appearance skin color (Appearance), and respiration (Respiration).

### 2.3. Composition detection of breast milk

On the morning of the 42nd day after delivery, 5 mL of breast milk from the single breast of the mother was collected with an electric breast pump or manual milking. The breast milk collected is the mid-stream breast milk (5–7 min after lactation). After recording the collection time, freeze it immediately and transfer it to the refrigerator at −80°C as soon as possible for further inspection.

Lactose, fat, protein, energy, minerals and water in breast milk were detected by infrared spectral analysis technology with an automatic breast milk composition analyzer (HKANGYU KY-9003).

### 2.4. Fecal sample collecting and 16S rRNA gene sequencing

Within postnatal days 35–42, the participants provided samples of infant feces using sterile stool collection tubes and transferred to the laboratory as soon as possible for storage in a −80°Crefrigerator.

Stool microbiome DNA was extracted according to the instructions of the Omega E.Z.N.A. Stool DNA Kit (MoBio Laboratories, Carlsbad, CA). The extracted DNA was tested for DNA quality and concentration by 1% agarose gel electrophoresis and NanoDrop 2000 spectrophotometry (Thermo Scientific Inc., United States). The quality-checked samples were stored at −20°C for subsequent experiments. V3-V4 regions of the 16S rRNA gene were amplified with the primers 338F (5′-ACTCCTACGGGAGGCAGCAG-3′) and 806R (5′-GGACTACHVGGGTWTCTAAT-3′) and then sequenced using the Illumina MiSeq sequencing platform (Illumina, San Diego, CA) at Beijing Ovison Gene Technology Co. Raw data was filtered to remove sequences less than 230 bp in length, and chimeric sequences were removed by comparing with the Gold Database database using the uchime method (10). OTU clustering (operational taxonomic units) of high-quality sequences was performed using the VSEARCH (v2.7.1) software uparse algorithm with a sequence similarity threshold of 97% (11). Comparison with the Silva128 database using the RDP Classifier algorithm was performed, and a confidence threshold of 70% was set to obtain the species classification information corresponding to each OTU.

### 2.5. Statistical analysis

SPSS (v23.0) was used for statistical analysis of infant characteristics, nutrient components of breast milk and clinical data. The α-diversity index analysis (including Shannon and Simpson indices) was performed using QIIME1 (v1.8.0) software. Based on species annotation and relative abundance results, species composition histogram analysis was performed using R Studio software (12). The beta diversity indices were also calculated using R Studio software. LEfSe (linear discriminant analysis effect size) was used to discover the features contributing to the most variation between control and case groups [LDA (linear discriminant analysis) > 2.0]. Phylogenetic Investigation of Communities by Reconstruction of Unobserved States (PICRUSt) was used to predict the functional composition of the microbial community metagenome from its 16S profile. The KEGG database was used to obtain KO, pathway and EC information. STAMP (v2.1.3) software package was used for analyzing metabolic profiles (13).

## 3. Results

### 3.1. Characteristics of population

In this study, 26 mother-infant pairs participants (13 LBMJ infants in the case group and 13 healthy individuals in the control group) were enrolled for questionnaire survey, breast milk composition determination and 16S rRNA gene sequencing.

Demographic characteristic information of the infants and their mothers is shown in Table 1. There were no significant differences in mother’s maternal age, BMI, gestational week, delivery mode, parity, pregnancy complications, infant’s gender, birth length, birth weight, 1 min Apgar score and 5 min Apgar score between the two groups.

**Table 1:** Demographic characteristic information of participants.

Variables		Case (*n* = 13)	Control (*n* = 13)	*p*-value
Maternal age (Mean ± SD, y)		31.62 ± 3.75	33.15 ± 3.13	0.268
BMI (Mean ± SD)		20.52 ± 2.39	21.64 ± 3.27	0.332
Gestational week (Mean ± SD, w)		38.98 ± 0.71	39.43 ± 0.57	0.089
Delivery mode (%)	Vaginal	10 (76.92)	12 (92.31)	0.277
	Cesarean	3 (23.08)	1 (7.69)	
Parity (%)	1	8 (61.54)	8 (61.54)	0.574
	2	5 (38.46)	4 (30.77)	
	3	0 (0.00)	1 (7.69)	
Pregnancy complications (%)	Yes	3 (23.08)	3 (23.08)	1
	No	10 (76.92)	10 (76.92)	
Gender (%)	Male	6 (46.15)	6 (46.15)	1
	Female	7 (53.85)	7 (53.85)	
Birth length (Mean ± SD, cm)		50.23 ± 2.09	50.54 ± 1.76	0.688
Birth weight (Mean ± SD, g)		3416.15 ± 447.07	3392.31 ± 400.96	0.887
1-min Apgar score (Mean ± SD)		9.92 ± 0.28	9.92 ± 0.29	1
5-min Apgar score (Mean ± SD)		9.92 ± 0.28	10.00 ± 0.00	0.337

### 3.2. Composition of breast milk

The composition of mother’s milk in case group and control group is shown in Table 2. In general, the nutritional components of breast milk in case group were lower than those in control group, but there were no significant statistical differences between the two groups.

**Table 2:** Nutrient components of breast milk.

Group	Lactose (g/100 g)	Fat (g/100 g)	Protein (g/100 g)	Energy (kcal/100 g)	Mineral (g/100 g)	Moisture content (g/100 g)
Case	7.38 ± 1.33	4.07 ± 1.44	0.90 ± 0.16	69.80 ± 9.31	0.22 ± 0.04	87.42 ± 1.04
Control	7.75 ± 1.38	4.26 ± 0.75	0.95 ± 0.17	73.10 ± 10.67	0.23 ± 0.04	86.81 ± 1.99

Mean ± SD, *n* = 13.

### 3.3. Clusters in infant gut microbiota

On an average 57,179 ± 25,385 reads were generated per sample and we identified 169 OTUs (operational taxonomic units). 169 OTUs were obtained from the two groups of subjects through cluster analysis, including 129 shared OTU sequences among two groups, 15 unique OTU sequences in case group and 25 unique OTU sequences in control group.

### 3.4. Fecal alpha and beta diversity

**Figure 1:**
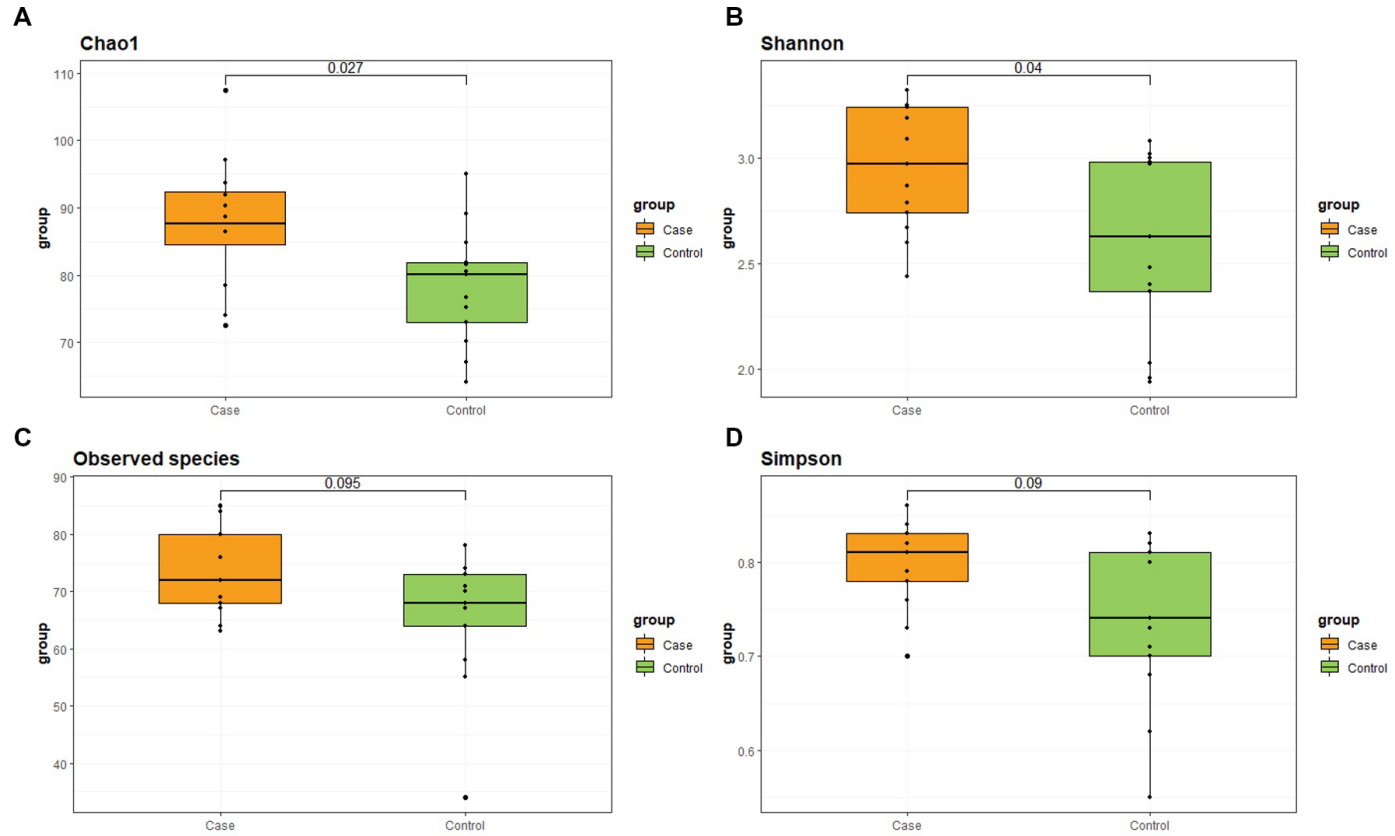
Differences of α-diversity between two groups. **(A)** Chao1 index. **(B)** Shannon index. **(C)** Observed species. **(D)** Simpson index.

The α-diversity indicators are shown in Figure 1 and Table 3. Chao1 and Shannon indexes have significant differences between the two groups, which are reflected in the higher α-diversity of case group.

**Table 3:** Alpha diversity index of two groups.

Group	Chao1	Observed_species	Shannon	Simpson
Case	87.59 (12.78)	72 (14.5)	2.97 (0.54)	0.81 (0.06)
Control	80.12 (11.76)	68 (12.5)	2.63 (0.79)	0.74 (0.13)

**Figure 2:**
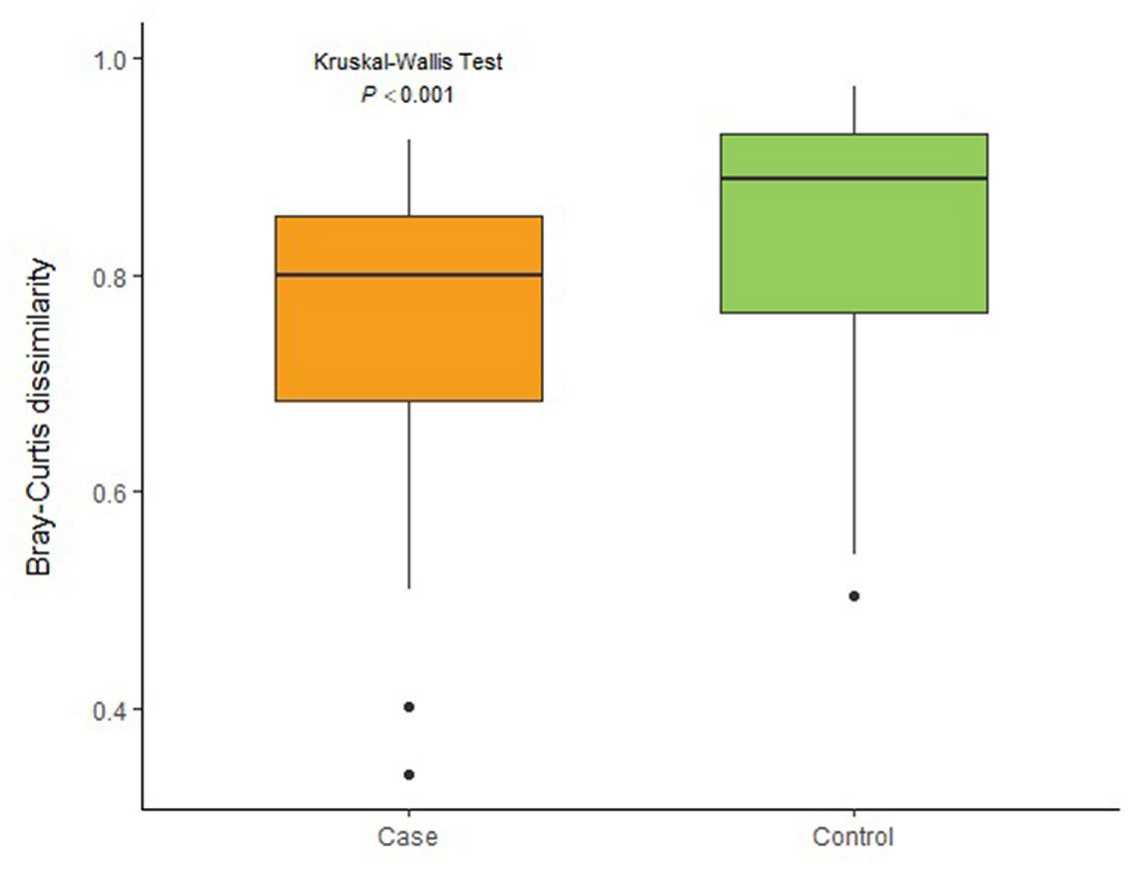
Differences of β-diversity between two groups.

To explore the diversity of clusters (β-diversity), the study calculated the community distance using Bray-Curtis distance based on the OTU counts. The Kruskal-Wallis’s test revealed a significant difference between the two groups in the diversity of the microbiota (Figure 2).

**Figure 3:**
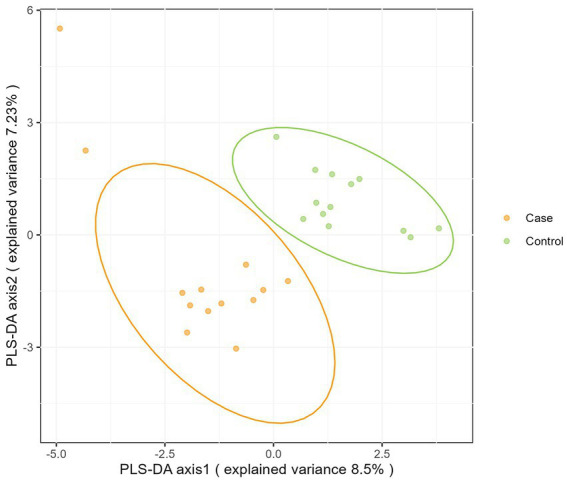
PLS-DA of two groups of bacteria based on OUT.

Moreover, as shown in Figure 3, microbiota from the two groups could be completely separated and significantly different (*p* < 0.05).

### 3.5. Taxonomic analysis

**Figure 4:**
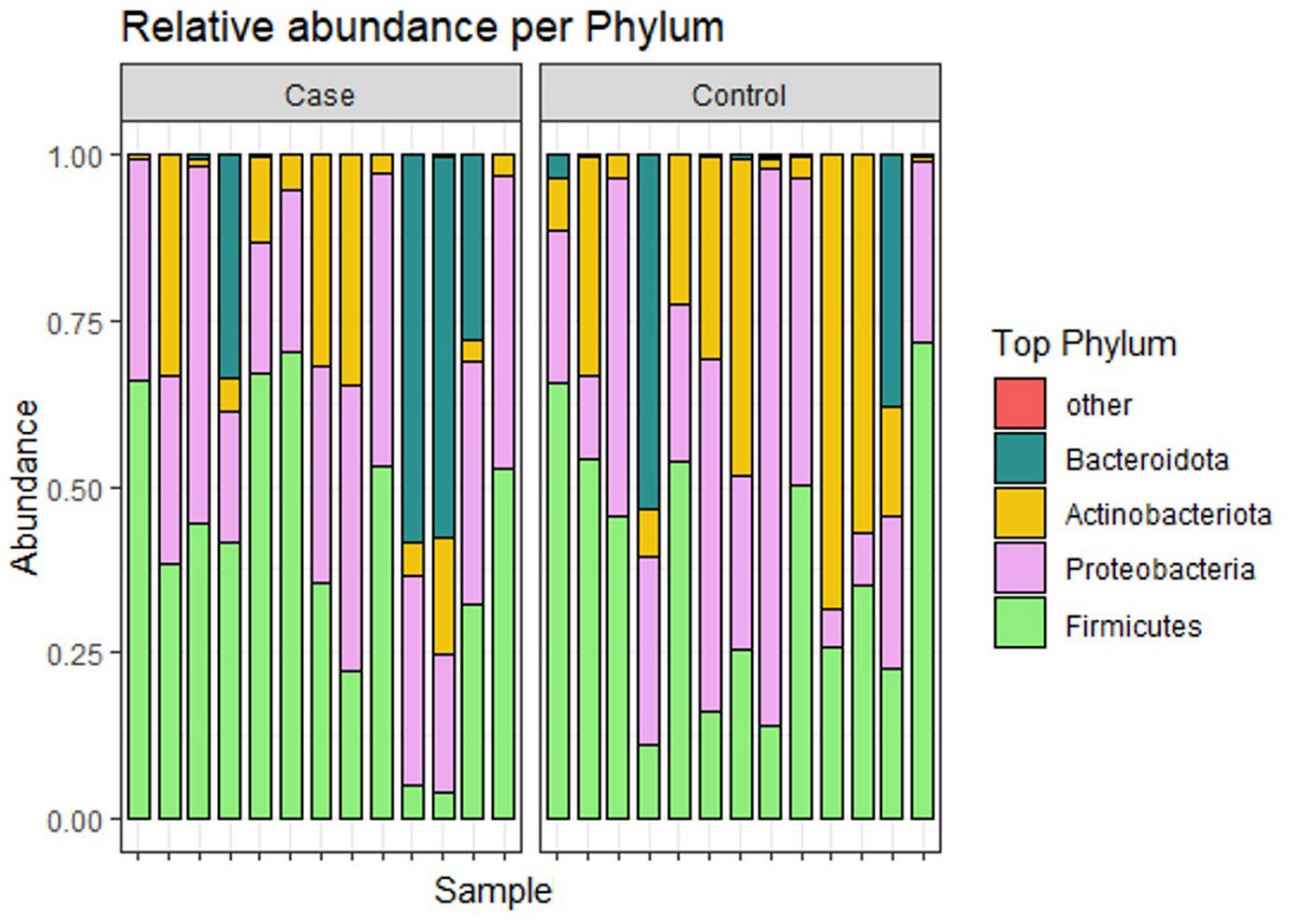
Relative abundance of microbiota at phylum level.

**Figure 5:**
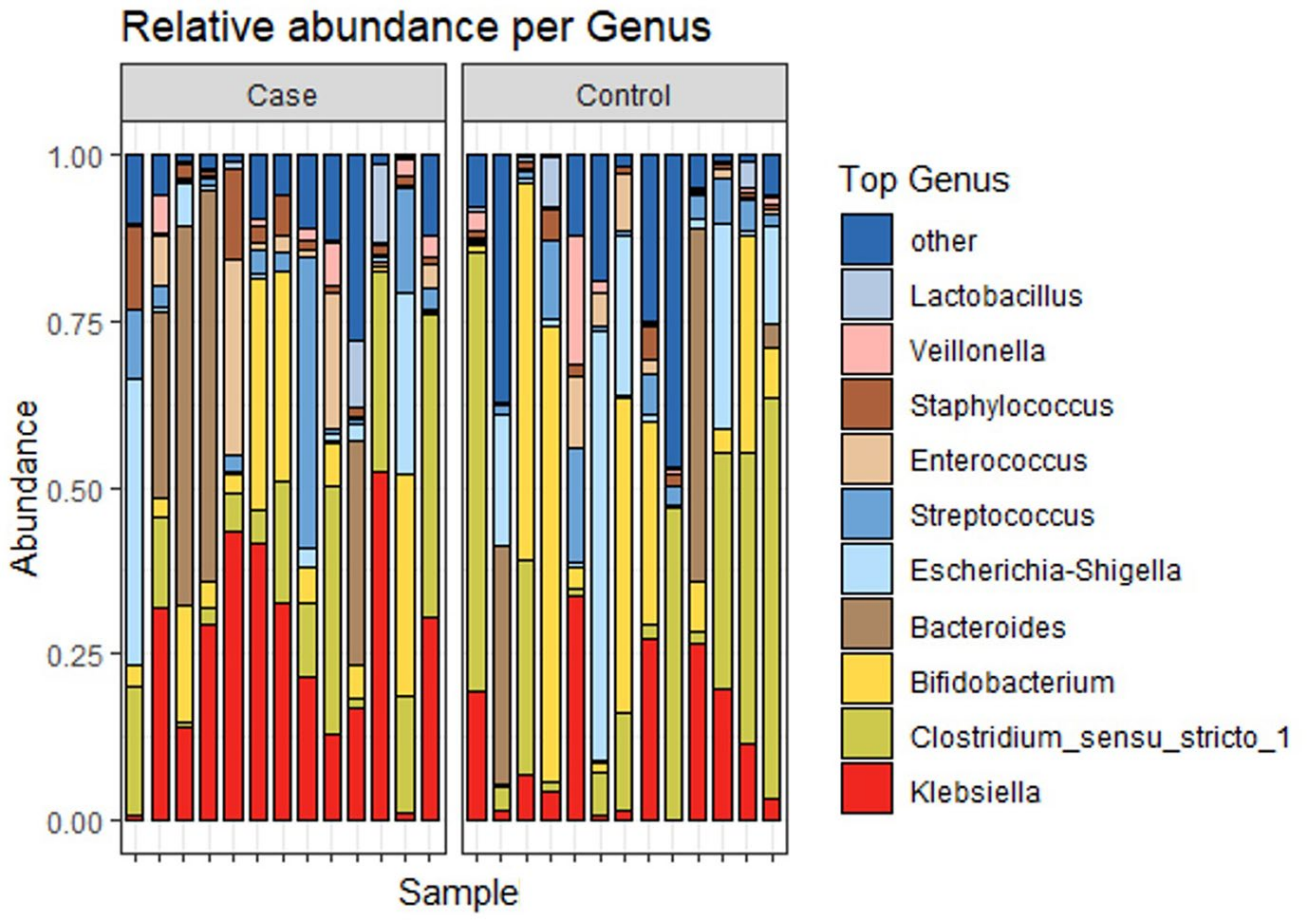
Relative abundance of microbiota at Genus level.

Figure 4 shows the relative abundance of the top 4 phyla of infant gut microbiota. The most dominant phylum among them is Firmicutes, accounting for 39.4% (41.0 and 37.8% in case and control groups, respectively). Other relatively abundant bacteria are Proteobacteria (33.2 and 31.6% in case and control groups, respectively), Actinobacteria (12.1 and 23.1% in case and control groups, respectively), and Bacteroidota (13.7 and 7.5% in case and control groups, respectively). Figure 5 shows the abundance of the top 10 genera of infant gut microbiota. At the genus level, we observed that *Klebsiella* is the most abundant genus in the study population, accounting for 18.7% (25.4 and 12.0% in case and control groups, respectively). In addition, the relative abundance of *Klebsiella* is significantly different between two groups (*p* < 0.05), the infants in the case group have a higher relative abundance. No significant difference was found in other bacteria.

We take the demographic information of the mother and infant, the composition information of breast milk, the relative abundance of each genus of bacteria and α-diversity index is used as independent variable, and LBMJ is used as dependent variable for stepwise regression analysis. After automatic identification of the model, the relative abundance of *Klebsiella* was finally left in the model, with the R square value of 0.197, which means that the relative abundance of *Klebsiella* could explain 19.7% of the change between groups. Moreover, the model passed the F test (*F* = 5.873, *p* = 0.023), indicating that the model was effective (Table 4). The model formula is: LBMJ = 0.224 + 1.477 * *Klebsiella*. In addition, it is found by testing the multicollinearity of the model that all VIF values in the model are less than 5, which means that there is no collinearity; The D-W value is near 2, which means that the model has no autocorrelation and no correlation between sample data, so the model is good. The value of *Klebsiella*’s regression coefficient is 1.477 (*t* = 2.423, *p* = 0.023), which means *Klebsiella* will have a significant positive relationship with jaundice.

**Table 4:** Spearman correlation analysis results.

	Coefficient	*t*	*P*	VIF	*R* ^2^	*F*
Constant	0.224	1.531	0.139	–	0.197	5.873
*Klebsiella*	1.477	2.423	0.023*	1.000		

D-W value: 2.398; *P < 0.05.

Furthermore, Spearman correlation coefficient was used to analyze the strength of the correlation between each genus and TcB value of forehead. The results showed that the correlation coefficient between TcB value and *Klebsiella* relative abundance was 0.392 (*p* < 0.05), indicating that there was a significant positive correlation between TcB and *Klebsiella* relative abundance.

**Figure 6:**
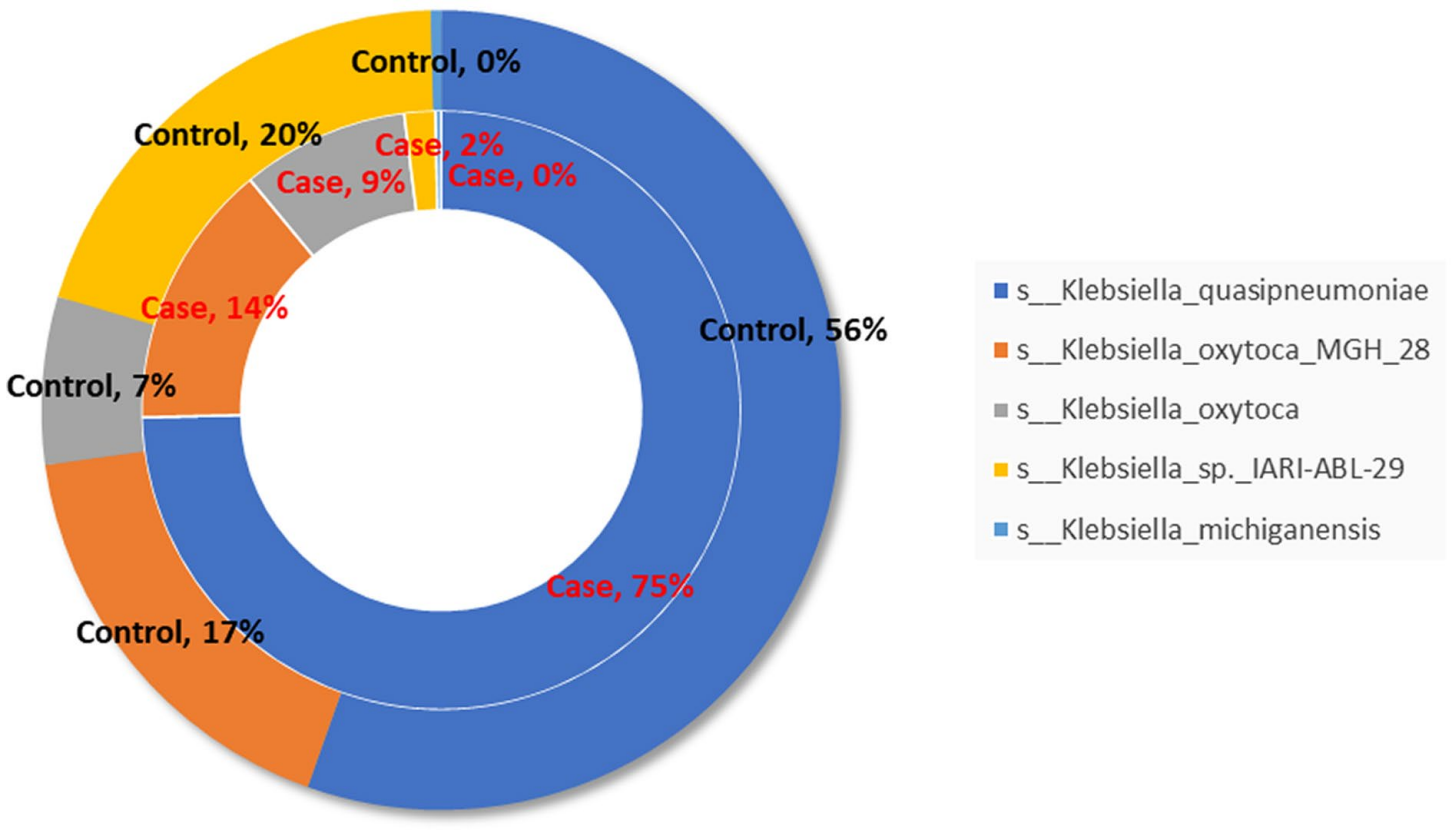
Species composition of *Klebsiella* genus of two groups.

The proportion of species belonging to *Klebsiella* genus is shown in Figure 6, in which *Klebsiella_quasipneumoniae* is dominant.

### 3.6. LEfSe analysis

**Figure 7:**
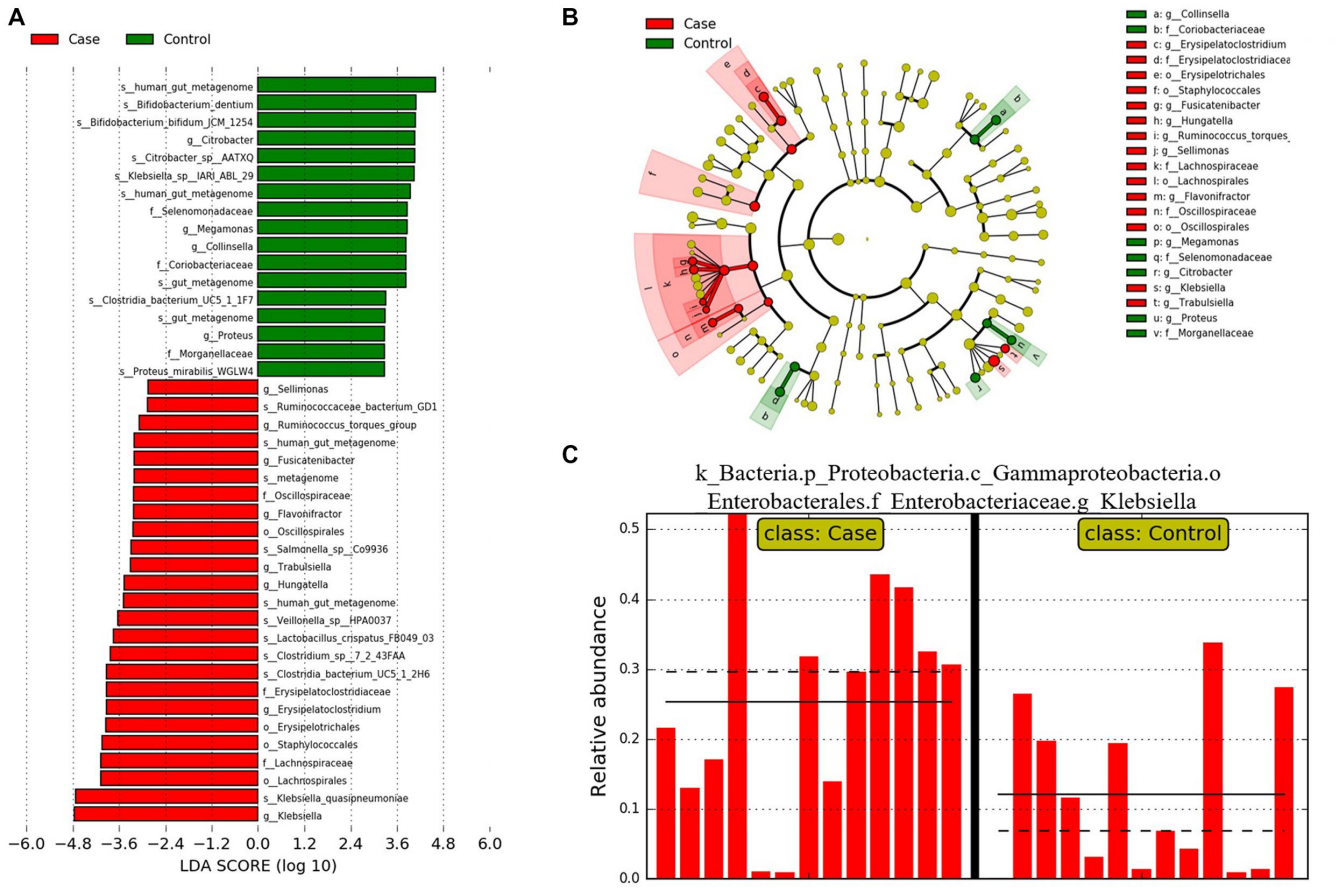
LEfSe analysis results of two groups. **(A)** Bar plot of the LDA Score between two groups. **(B)** Evolutionary branching diagram of LEfSe analysis based on taxonomic information between groups. **(C)** Histogram of the *Klebsiella* relative abundances between groups.

LDA Effect Size analysis (LEfSe) was employed to identify different features between control and case groups at all levels. According to the threshold LDA >2.0, 42 features were found to be significantly different between controls and cases. Twenty five features were more abundant in case group and 17 features was more abundant in the control group (Figure 7). The relative abundance of *Klebsiella*, which exhibited the highest LDA score, was significantly associated with jaundice.

### 3.7. PICRUSt 2 function prediction analysis

**Figure 8:**
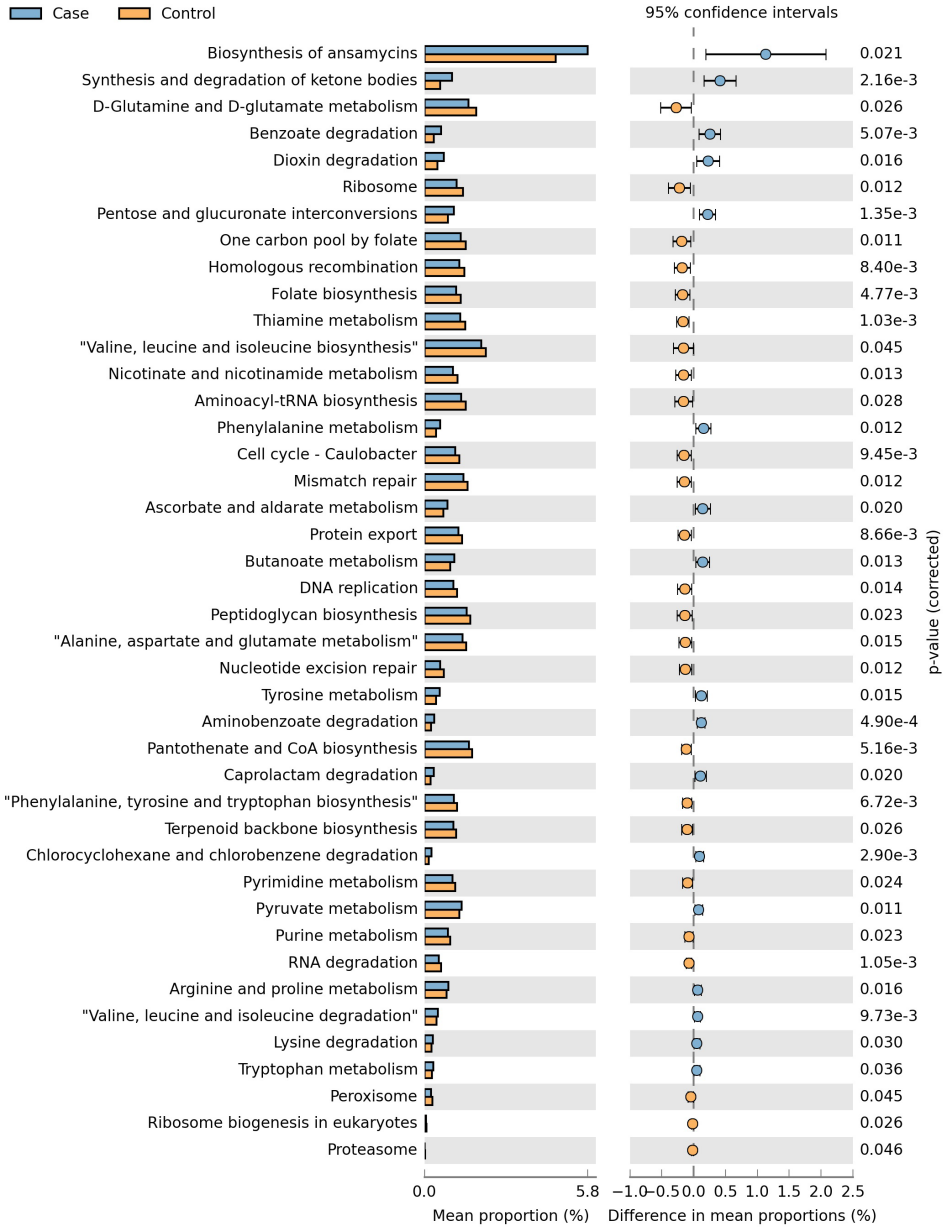
Metabolic pathways predicted to be related to the occurrence of breast milk jaundice.

Phylogenetic Investigation of Communities by Reconstruction of Unobserved States 2 (PICRUSt2) was used to predict microorganisms of the 16S rRNA data followed by function categorizing according to KEGG orthology. In the study, 42 metabolic pathways belonging to Metabolism, Genetic Information Processing and Environmental Information Processing were predicted to be associated with the presence of LBMJ (Figure 8).

## 4. Discussion

There are several different theories about the pathogenesis of LBMJ, which are still inconclusive. Jaundice is essentially due to an imbalance in the production and clearance of bilirubin. We collected breast milk and infant fecal sample for a comprehensive comparative analysis in an attempt to find a link with the development of LBMJ.

We compared the breast milk macronutrients between the case and control groups and no significant differences were found. However, a trend toward lower nutrient composition in the case group could be observed, a result similar to those found in other studies. It is suggested that ensuring the adequacy of nutrients in newborns may be beneficial in reducing their jaundice. This result is only a clue, and follow-up studies with larger sample sizes and more in-depth testing of breast milk composition are needed (14).

As an important part of the pathogenesis of neonatal jaundice, the enterohepatic circulation has attracted widespread attention. Changes in the gut microbiota may be one of the mechanisms leading to LBMJ. In this study, we found that the gut microbiota in LBMJ infants had unique features that can affect the occurrence and development of LBMJ. In total, 13 infants with LBMJ and 13 healthy infants were selected for intestinal microbiome composition analysis. High-throughput sequencing covered more than 99.84% of the genes, and the sequencing depth was sufficient to compare the richness of taxa.

Alpha-diversity provides the most fundamental statistic of microbial communities, higher α-diversity index means more species and more evenness (15). Chao1 index focuses on measuring richness, while Shannon index gives consideration to both rich-ness and evenness. There was found a higher richness/evenness in case group on Chao1 and Shannon index but no difference using another (Simpson). Generally speaking, higher diversity is considered to be more ecologically valuable and stable. The intestinal microbiota in LBMJ infants is characterized by a significant increase in species richness, possibly due to the proliferation of potentially pathogenic species (16). In this study, the case group showed higher diversity, which may be related to the presence of more pathogenic bacteria, but this requires more research to verify.

At the phylum level, no significant difference in the relative abundance of the two groups was observed. However, at the genus level, the dominant genus *Klebsiella* was significantly elevated in the case group, more than half of which were *Klebsiella quasipneumoniae* species (17). LEfSe analysis also showed that *Klebsiella* was the most significant bacteria in the case group. *Klebsiella* is widespread in the environment, and is frequently linked to an abnormal pattern of intestinal microbiota (18). *Klebsiella_quasipneumoniae* belongs to the *Klebsiella pneumoniae species* complex, which can pose a serious health threat to newborns and immunocompromised (19). In a study characterizing the gut microbiota of 29 healthy Chinese neonates and 2-month-old infants, *Escherichia/Shigella* and *Klebsiella* were the main genera of Enterobacteriaceae in Chinese neonates (20). And the enrichment of *Enterobacteriaceae* in the gut is usually associated with the pathogenesis of cholecystitis and IBD (inflammatory bowel disease) (21). Therefore, these abundant *Klebsiella* may pose a health threat to infants.

In the first few days after birth, the microbiota in the intestine has not been established, so the conjugated bilirubin that enters the intestine with bile cannot be reduced to fecal bilirubin; On the other hand, there are more β- glucuronidase in the intestine of new-born can hydrolyze conjugated bilirubin into unconjugated bilirubin, which is absorbed by intestinal mucosa and returned to liver through portal vein (22). This process of enterohepatic circulation in neonates cause the burden of liver metabolism of bilirubin increases, and more unconjugated bilirubin remains in the blood.

β-glucuronidase participates in the decomposition of bilirubin and plays a role in the development of hyperbilirubinemia in infants. Studies have found that the intestinal metagenome of infants at birth and 12 months old has a higher level of β-glucuronidases than that of their mothers (23). Later research found that, many bacteria, such as *E. coli*, *Klebsiella* sp., *Clostridium* can possess β-glucuronidase activity (24). Therefore, we have reason to speculate that *Klebsiella* in the study may be a key participant in the production of intestinal microbial β-glucuronidase, which further induces hyperbilirubinemia.

Beta-diversity can be used to measure between-group differences in microbiota communities. The Kruskal-Wallis’s test showed a difference in the bacterial composition profiles between case and control groups. Moreover, PLS-DA analysis revealed that participants in case group cluster together in multidimensional space by their micro-biota, but separately from control group (25). These results all prove that the intestinal microbiota structure of infants in the case group is significantly different from that of normal infants.

Functional prediction analysis screened 42 metabolic pathways that may be related to the occurrence of LBMJ. Among them, Ansamycin synthesis has the greatest impact in case group. Ansamycin is a spiro piperidine derivative of rifamycin (26). It is mainly used for pulmonary infection of mycobacterium, and effective for rifampicin resistant *mycobacterium tuberculosis* strains. Its adverse reactions are similar to rifampicin, so the exposure is also positively related to the concentration of bilirubin (27). However, such antibiotics should significantly inhibit *Klebsiella*, which is inconsistent with the results of this study and is worth for further exploration (28). Functional prediction results can also be used as a reference for future research.

This study has two strengths. First, breast milk composition has the potential to influence the development of jaundice and the composition of the intestinal microbiota. We determined the macronutrients composition of breast milk and obtained some preliminary trends that set the stage for further studies. Secondly, we analyzed the correlation between different bacteria and bilirubin levels, which also helps to determine the relationship between intestinal microbiota and jaundice. However, there are two shortcomings in this study. One is that the amino acid composition and fatty acid composition of breast milk were not examined and analyzed, so that more information on the nutritional composition of breast milk and its effect on the development of intestinal microbiota and jaundice could not be obtained. Second, our study was a characteristics analysis of intestinal microbiota profiles based on a cross-section study, so the conclusions drawn could not be further causally inferred. In the future, as the size of mother-infant cohort expands, we will test and refine these findings by expanding the sample size and analyzing infant gut microbiota at multiple points in time.

## 5. Conclusion

In conclusion, the richness and diversity of intestinal microbiota were significantly different in the case group compared to the control group. The important biomarker *Klebsiella* with significant difference was found through stepwise regression and LEfSe analysis. Combining the correlation analysis of *Klebsiella*’ relative abundance and TcB value, it is found that *Klebsiella* is closely related to the disease severity of LBMJ patients. A total of 42 genera showed statistically significant differences between the two groups. Functional prediction analysis of 16S indicated that 42 metabolic pathways may be related to the occurrence of LBMJ.

## Data availability statement

The data presented in the study are deposited in the NCBI repository, accession number PRJNA951195.

## Ethics statement

The studies involving human participants were reviewed and approved by the Ethics Committee of the Peking University People’s Hospital (Approval Number: 2020PHB113-01). Written informed consent to participate in this study was provided by the participants’ legal guardian/next of kin.

## Author contributions

QG and XRL: design and guidance of the trial. QG: methodology. XRL, MC, XNL, and SZ: sample collection. QG, XRL, LP, and XP: data curation. QG and XRL: writing—original draft preparation. LW and PL: writing—review and editing. PL: funding acquisition. All authors agreed to be accountable for the content of the work.

## Funding

This research was supported by the construction of breast milk bank based on birth cohort (2020-Z-19) grant funded by the Ausnutria Maternal and Child Nutrition Research Fund (AU-YJY-B-LX-19-018) and Hunan Engineering and Technology Research Center for Nutrition and Health Products, Innovation Platform and Talent Plan (2019TP2066).

## Conflict of interest

LP and XP were employed by the Ausnutria Dairy (China) Co., Ltd.

The remaining authors declare that the research was conducted in the absence of any commercial or financial relationships that could be construed as a potential conflict of interest.

## Publisher’s note

All claims expressed in this article are solely those of the authors and do not necessarily represent those of their affiliated organizations, or those of the publisher, the editors and the reviewers. Any product that may be evaluated in this article, or claim that may be made by its manufacturer, is not guaranteed or endorsed by the publisher.
